# Passive targeting of thermosensitive diblock copolymer micelles to the lungs: synthesis and characterization of poly(*N*-isopropylacrylamide)-*block*-poly(ε-caprolactone)

**DOI:** 10.1186/s12951-015-0103-7

**Published:** 2015-06-18

**Authors:** Ren-Shen Lee, Chih-Hung Lin, Ibrahim A Aljuffali, Kai-Yin Hu, Jia-You Fang

**Affiliations:** The Center of General Education, Chang Gung University, Kweishan, Taoyuan, Taiwan; Center for General Education, Chang Gung University of Science and Technology, Kweishan, Taoyuan, Taiwan; Department of Pharmaceutics, College of Pharmacy, King Saud University, Riyadh, Saudi Arabia; Pharmaceutics Laboratory, Graduate Institute of Natural Products, Chang Gung University, 259 Wen-Hwa 1st Road, Kweishan, Taoyuan, 333 Taiwan; Immunology Consortium, Chang Gung Memorial Hospital, Kweishan, Taoyuan, Taiwan; Research Center for Industry of Human Ecology, Chang Gung University of Science and Technology, Kweishan, Taoyuan, Taiwan

**Keywords:** Copolymer, Micelle, Thermosensitivity, Carboplatin, Lung, Passive targeting

## Abstract

**Background:**

Amphiphilic poly(*N*-isopropylacrylamide)-*block*-poly(ε-caprolactone) (PNiPAAm-*b*-PCL) copolymers were synthesized by ring-opening polymerization to form thermosensitive micelles as nanocarriers for bioimaging and carboplatin delivery.

**Results:**

The critical micelle concentration increased from 1.8 to 3.5 mg/l following the decrease of the PNiPAAm chain length. The copolymers revealed a lower critical solution temperature (LCST) between 33 and 40°C. The copolymers self-assembled to form spherical particles of 146–199 nm in diameter. Carboplatin in micelles exhibited a slower release at 37°C relative to that at 25°C due to the gel layer formation on the micellar shell above the LCST. The micelles containing dye or carboplatin were intravenously injected into the rats for in vivo bioimaging and drug biodistribution. The bioimaging profiles showed a significant accumulation of micelles in the lungs. The micelles could minimize the reticuloendothelial system (RES) recognition of the dye. In vivo biodistribution demonstrated an improved pulmonary accumulation of carboplatin from 2.5 to 3.4 μg/mg by the micelles as compared to the control solution. Carboplatin accumulation in the heart and kidneys was reduced after encapsulation by the micelles.

**Conclusion:**

This study supports the potential of PNiPAAm-*b*-PCL micelles to passively target the lungs and attenuate RES uptake and possible side effects.

**Electronic supplementary material:**

The online version of this article (doi:10.1186/s12951-015-0103-7) contains supplementary material, which is available to authorized users.

## Background

Polymeric micelles are the nanoparticles developed by the self-assembly of amphiphilic polymers. They are useful for entrapping lipophilic drugs for targeted anticancer drug delivery via the enhanced permeation retention (EPR) effect. The micelles are also found to prolong the circulation time of drugs, reduce the adverse effect, and improve the therapeutic index [1, 2]. Polymeric micelles/nanoparticles are the biomedical nanostructures with the most commercialized products after liposomes [3]. Biodegradable polymers are preferred as the nanomaterials for therapeutic application. Poly(ε-caprolactone) (PCL) is one of the biodegradable polymers widely used in nanoparticles for bioimaging, drug delivery, and tissue scaffolding [4]. This polyester is approved by the US Food and Drug Administration (USFDA) because of its biocompatibility, biodegradation, and nontoxicity in nature [5]. Due to its lipophilic characteristics, pendent functionalization with other polymers to produce the copolymers is highly needed to achieve the suitable hydrophilicity, bioadhesion, and drug encapsulation for biological use [6].

Poly(*N*-isopropylacrylamide) (PNiPAAm) is a thermosensitive polymer with a lower critical solution temperature (LCST) of ~32°C. It is extensively employed to form the temperature-responsive copolymers with other polymers [7]. Some investigations synthesize the copolymers of PNiPAAm and PCL conjugates for their biocompatibility and temperature sensitivity [6, 8, 9]. For temperature-triggered drug delivery, it is generally recognized that the thermosensitive micelles show a slow drug release at temperatures below the LCST, whereas heating above the LCST results in the destruction of the micellar structure and the expulsion of the entrapped drug. However, in the case of the conjugates of PNiPAAm and PCL, a gel layer on the micellar shell is formed after triggering the thermoresponsiveness [10, 11]. This layer creates a diffusion barrier to enhance the sustained drug release.

Although the copolymers related to combined PNiPAAm and PCL were developed previously, no in vivo data proved their usefulness for biological application. Herein we blocked the lipophilic segments of PCL to the hydrophilic block of PNiPAAm to prepare a core–shell micelle. We aimed to develop a multifunctional nanocarrier for bioimaging and controlled drug delivery. The diblock copolymers were synthesized with different PNiPAAm and PCL block lengths. The micelles were fabricated using a dialysis technique to determine the physicochemical properties. Carboplatin was used as the model anticancer drug in this study. The passive targeting of the micelles to organs was examined. The safety of the micelles was evaluated at the cell level, including the lactic dehydrogenase (LDH) leakage of human neutrophils and the viability of 293T and HaCaT cells.

## Results

### Synthesis of PNiPAAm-b-PCL

**Figure 1:**
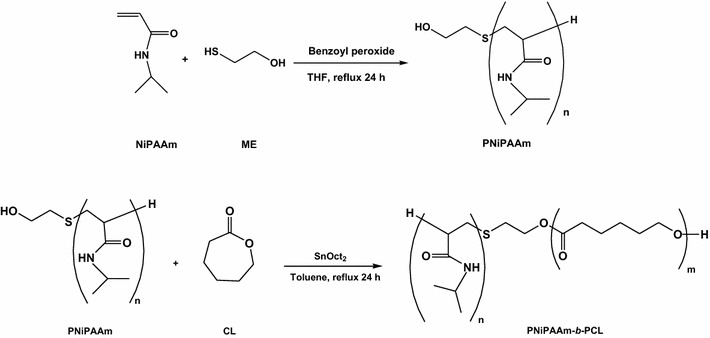
Synthetic procedures of PNiPAAm and PNiPAAm-*b*-PCL.

**Figure 2:**
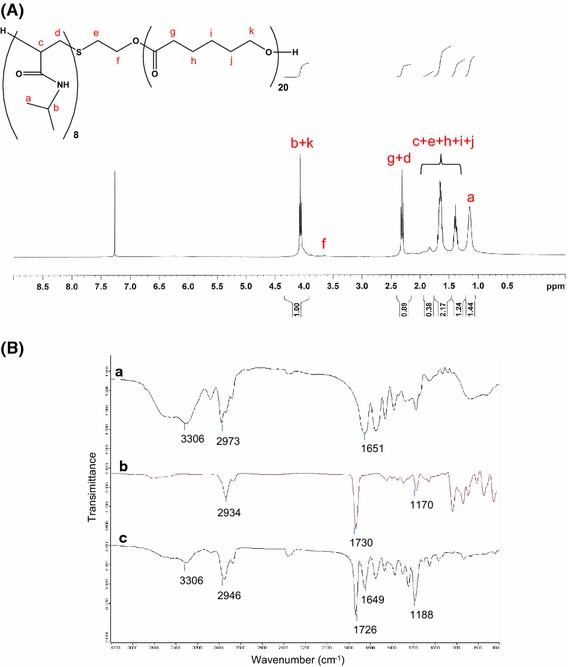
The representative ^1^H NMR spectrum of PNiPAAm8-*b*-PCL20 (**A**) and FTIR spectra of PNiPAAm8 (*line a*), CL (*line* *b*) and PNiPAAm8-*b*-PCL20 (*line c*) (**B**).

PNiPAAm-*b*-PCL diblock copolymers were synthesized by ring-opening polymerization of CL with hydroxyl-terminated PNiPAAm as shown in Figure 1. Three copolymers with different monomer numbers, including PNiPAAm8-*b*-PCL20, PNiPAAm9-*b*-PCL15, and PNiPAAm14-*b*-PCL59, were conjugated in this work. Figure 2A illustrates the representative ^1^H NMR spectrum of PNiPAAm8-*b*-PCL20. Observations showed typical signals of PNiPAAm blocks at 1.15 ppm (H_a_, –CH(CH_3_)_2_), 3.88–4.08 ppm (H_b_, –CH(CH_3_)_2_), 1.35–1.83 ppm (H_c_, –CH_2_–CH–), and 2.15–2.33 ppm (H_d_, CH_2_–CH–). The signals at 2.15–2.33 ppm were also assigned to the protons of PCL (H_g_, –COCH_2_–). The chemical shifts at 1.35–1.83 ppm were ascribed to the protons of PCL (H_h_, H_i_, and H_j_, –CH2–). The peaks at 3.88–4.08 ppm corresponded to –OCH_2_– moiety in PCL (H_k_).

Figure 2B displays the FTIR spectra of PNiPAAm8, CL, and the grafted PNiPAAm8-*b*-PCL20. The PNiPAAm spectrum showed the characteristic peaks at 3,306 cm^−1^ (N–H), 2,973 cm^−1^ (C–H), and 1,651 cm^−1^ (amide C=O). The CL spectrum revealed the signals at 2,934 cm^−1^ (C–H), 1,730 cm^−1^ (ester C=O), and 1,170 cm^−1^ (C–O). In the FTIR spectrum of the grafted copolymer, the existence of the peaks from PNiPAAm and CL confirmed an effective synthesis of PNiPAAm-*b*-PCL. The absorption peak at 1,188 cm^−1^ became stronger in the spectrum of the copolymer as compared to that of CL, indicating that there existed a number of C–O groups introduced by PCL backbone. The molecular weights of the diblock copolymers are measured by GPC as shown in Additional file 1: Table S1. The molecular weight of PNiPAAm8-*b*-PCL20, PNiPAAm9-*b*-PCL15, and PNiPAAm14-*b*-PCL59 was 5,578, 3,792, and 6,879 Da, respectively. The traces of GPC exhibited a unimodal peak, demonstrating a narrow molecular weight distribution of the copolymers.

### Characterization of PNiPAAm-b-PCL micelles

**Figure 3:**
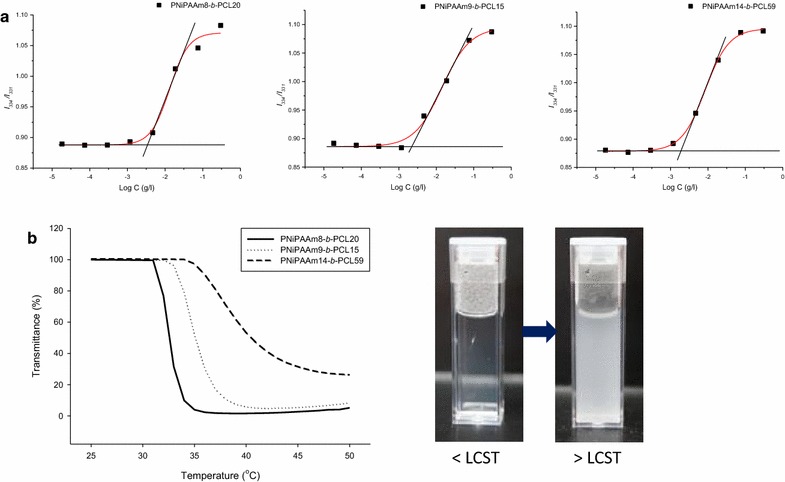
Plots of *I*_334_/*I*_331_ intensity ratio versus the logarithm of copolymer concentration for PNiPAAm-*b*-PCL (**a**) and phase transition curves (**b**) of PNiPAAm-*b*-PCL from 25 to 50°C.

The excitation spectra of pyrene in copolymer solution were examined to calculate the values of the critical micelle concentration (CMC). The distinguishing property of the excitation spectra was a red shift from 331 to 334 nm upon pyrene partitioning into the lipophilic core of the micelles. Figure 3 depicts the intensity ratio (*I*_334_/*I*_331_) of pyrene versus the logarithm of the copolymer concentration (log C). The *I*_334_/*I*_331_ ratio showed a substantial increase at a particular concentration, suggesting the pyrene entrapment into the micelles. Additional file 1: Table S1 summarizes the CMC values of different copolymers. It could be observed that the CMC decreased following the increase of the PNiPAAm chain length. The copolymer with the longest PCL length (PNiPAAm14-*b*-PCL59) demonstrated the lowest CMC (1.75 mg/l) among the copolymers.

We had characterized the LCST of the copolymer micelles by recognizing the transmittance change. As shown in the left panel of Figure 3b, a sharp transmittance reduction is visualized at a determined temperature. This indicates a phase transition from a transparent and clear solution to a formation of suspension containing aggregates as observed in the right panel of Figure 3b. As shown in Additional file 1: Table S1, 33, 35, and 40°C, respectively, are the LCST of PNiPAAm8-b-PCL20, PNiPAAm9-b-PCL15, and PNiPAAm14-b-PCL59.

Additional file 1: Table S1 summarizes the size, polydispersity, and zeta potential of the micelles. At 25°C, PNiPAAm8-*b*-PCL20 revealed a mean diameter of 146 nm. The average size was increased following the increase of the PNiPAAm chain length. The polydispersity of the three micelles ranged between 0.22 and 0.25, expressing a narrow size distribution. The zeta potential of the micelles was anionic, with no significant difference among the surface charge of the three nanosystems. The negative zeta potential of the micelles is a result of lone-pair electrons in the grafted PNiPAAm.

### Stability of PNiPAAm-b-PCL micelles

**Figure 4:**
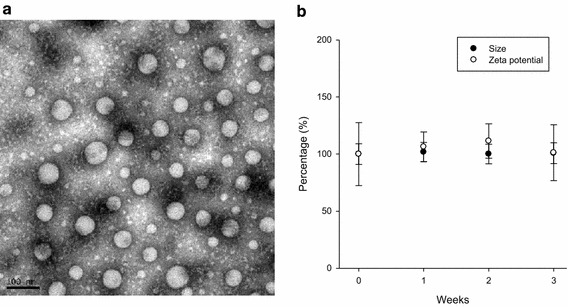
Transmission electron microscopic micrograph of PNiPAAm8-*b*-PCL20 micelles (**a**) and storage stability of PNiPAAm8-*b*-PCL20 micelles determined by average size and zeta potential (**b**). Each value represents the mean ± SD (*n* = 4).

PNiPAAm displays a coil-to-globule transition temperature at 32°C, which can undergo a conversion from room temperature to body temperature (37°C). This LCST is feasible for clinical application. PNiPAAm8-*b*-PCL20 was selected as the model copolymer for further investigation because its LCST (33°C) is so close to 32°C. Figure 4a illustrates the TEM image of the PNiPAAm8-*b*-PCL20 micelles. A spherical core–shell morphology was observed. The self-assembled micelles were well distributed as individual particles. The micellar size shown in the TEM image was smaller than that determined by the laser-scattering technique. A broad size range was also observed by TEM. This is attributed to the detection of the dehydrated solid state by TEM, whereas a hydrodynamic diameter was detected by laser scattering. The micelles were water-swollen in the hydrodynamic state.

The stability of the PNiPAAm8-*b*-PCL20 micelles was examined by incubating the nanosystems at 25°C for 3 weeks. Average diameter and zeta potential were used as the indicators to evaluate the micellar stability. As illustrated in Figure 4b, the size and zeta potential of PNiPAAm8-*b*-PCL20 showed no significant change during the 3 weeks. This suggests that the thermosensitive micelles are stable for at least 3 weeks; thus further application can be promised.

### The change of physicochemical properties of micelles as a function of temperature

The size and zeta potential of PNiPAAm8-*b*-PCL20 were assessed by varying the temperature from 25 to 40°C. As shown in Additional file 1: Table S2, the diameter of the micelles increased from 146 to 370 nm when the temperature was raised from 25 to 36°C. A further size augmentation to 567 nm was found as the temperature was increased to 40°C. The polydispersity of the micelles at 40°C was 1.00, indicating a very broad distribution of the different sizes in the nanosystems. The zeta potential remained constant after the heating.

### Carboplatin release from micelles

**Figure 5:**
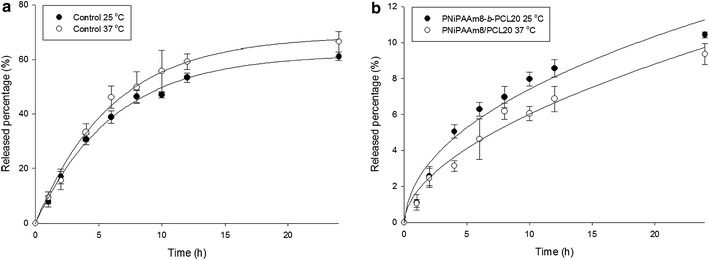
In vitro cumulative amount-time profiles across cellulose membrane of carboplatin release from control solution (**a**) and PNiPAAm8-*b*-PCL20 micelles (**b**) assessed by Franz diffusion cell at 25 and 37°C. Each value represents the mean ± SD (*n* = 4).

To explore the effect of temperature on carboplatin delivery, the drug-release experiment was performed at the temperatures of 25 and 37°C. Figure 5 depicts the percentage of released carboplatin plotted as a function of time. A 4% DMSO in double-distilled water was used as the control vehicle for dissolving carboplatin. As shown in Figure 5a, a two-phase release pattern was observed for carboplatin released from the control solution. Carboplatin exhibited an initial burst from the control, then gradually leveled off after 10 h. About 65% of the carboplatin had been released by 24 h. The control solution showed a faster release of carboplatin above the LCST than below the LCST. This discrepancy was not significant. Encapsulation of carboplatin into PNiPAAm8-*b*-PCL20 micelles revealed a more controllable drug release compared to the control as observed in Figure 5b. The burst carboplatin release was alleviated by the micellar incorporation. Carboplatin was continuously released from the micelles within 24 h. Approximately 10% of the carboplatin was released at the end of the experiment (24 h). When the temperature was elevated from 25 to 37°C, the drug release was suppressed to a significant degree.

### Cytotoxicity of the micelles

**Figure 6:**
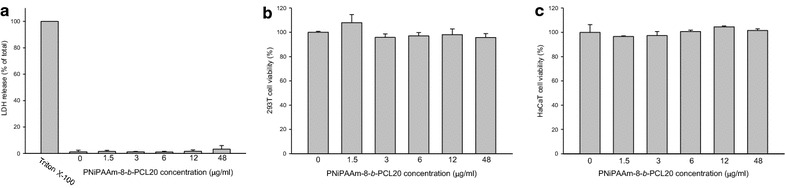
The LDH release of neutrophils (**a**) and the cell viability (%) of 293T cells (**b**) and HaCaT cells (**c**) treated by PNiPAAm8-*b*-PCL20 micelles. Each value represents the mean ± SD (*n* = 3).

The preliminary examination on the cytotoxicity of PNiPAAm8-*b*-PCL20 was performed in various cells. Figure 6 shows the results. Neutrophils are the main leukocytes (50–70%) in systemic circulation. The cytotoxicity of neutrophils can be associated with disruption of membrane integrity as indicated by LDH. As demonstrated in Figure 6a, micelle intervention does not show cytotoxicity against human neutrophils at all concentrations tested (1.5–48 μg/ml). Human embryonic kidney cells (293T) and keratinocytes (HaCaT) were chosen as the model systems to evaluate the cytotoxicity of micelles. Figure 6b, c exhibit the cell viability (%) of 293T and HaCaT in the presence of micelles, respectively. The copolymer micelles did not elicit a significant cytotoxicity to both cell lines compared to the nontreatment control.

### Organ bioimaging

**Figure 7:**
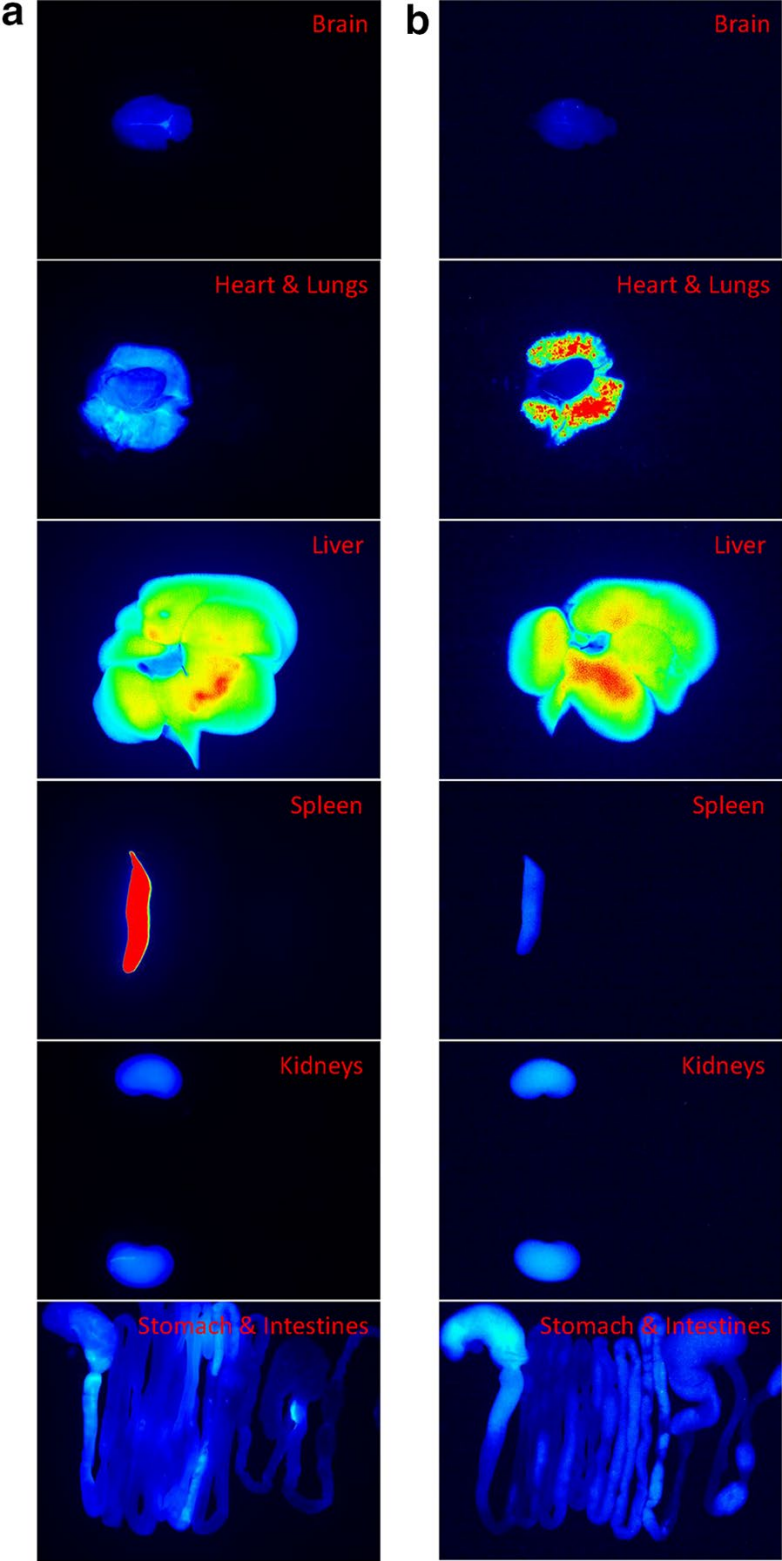
Fluorescence imaging of organs under Pearl^®^ Impulse system at near IR wavelength after a 2-h injection of DiR control solution (**a**) and DiR-loaded PNiPAAm8-*b*-PCL20 micelles (**b**).

The possible application of the copolymer micelles was further assessed by organ bioimaging and drug distribution in rats. The rats intravenously administered with DiR solution or micelles were sacrificed 2 h postinjection, and the organs were then harvested and imaged as shown in Figure 7. DiR solution clearly resided in the reticuloendothelial system (RES), including the liver and spleen (Figure 7a). Some signals were shown in the brain, lungs, kidneys, and gastrointestinal tract. However, the intensity of these organs was much less than that of RES. The bright signal of the liver was also achieved by micelle administration (Figure 7b). A negligible signal was visualized in the spleen treated with micelles, indicating a lower RES uptake of micelles than the control. A lower level of intensity was also noted in the brain treated with micelles compared to DiR solution. It is noticeable that, in contrast to the control, the micelles induced a much greater accumulation in the lungs. An extensive distribution of near IR signal was observed in the lungs treated with micelles. The near IR intensity in the kidneys and gastrointestinal tract was comparable between the two groups.

### Biodistribution of carboplatin

To investigate the delivery effectiveness of the micelles, in vivo biodistribution of carboplatin in the organs of interest was assessed. Additional file 1: Table S3 compares carboplatin accumulation in the organs at 2 h following intravenous injection of the control solution and micelles. The carboplatin uptake by the brain, liver, and spleen was comparable between the aqueous solution and the micelles. For the PNiPAAm8-*b*-PCL20 micelles, 3.35 μg/mg carboplatin was distributed in the lungs, which was a higher accumulation (*p* < 0.05) than in the control (2.5 μg/mg). Inclusion within the micelles allowed a significantly lower drug heart uptake (1.04 μg/mg) compared to the control (1.83 μg/mg) (*p* < 0.05). Lower carboplatin concentration (*p* < 0.05) in the kidneys was detected at 2 h postinjection of micelles (0.74 μg/mg) compared to aqueous solution (1.45 μg/mg).

## Discussion

Polymeric modification by copolymer strategy is advantageous for biomedical use because of the possibility of modulating drug release, mechanical properties, and biocompatibility [12]. We had synthesized PNiPAAm-*b*-PCL diblock copolymers consisting of thermosensitive moiety and biodegradable polyester backbones. PNiPAAm-*b*-PCL formed self-assembly micelles containing a core made from PCL blocks surrounded by PNiPAAm thermoresponsive coronal chains. The spectra of ^1^H NMR and FTIR confirmed a successful coupling between PNiPAAm and PCL.

The copolymer with the largest PCL block (PNiPAAm14-*b*-PCL59) showed the lowest CMC. This could be due to the stronger lipophilic interaction and reduced solvency resulting from the more lipophilic core in the presence of long PCL chains [13]. Previous studies [14, 15] also suggest that the increased lipophilic segment or decreased hydrophilic segment in PNiPAAm-based copolymers resulted in the lower CMC. However, this concept cannot explain the higher CMC of PNiPAAm8-*b*-PCL20 compared to PNiPAAm9-*b*-PCL15 since the former copolymer possessed less hydrophilic moiety and more lipophilic moiety than the latter copolymer. This could be due to the greater molecular weight of PNiPAAm8-*b*-PCL20 than PNiPAAm9-*b*-PCL15. The high molecular mass of the copolymer may lead to the high threshold for forming micelles. The experimental results indicate that PNiPAAm-*b*-PCL exhibited a lower CMC as compared to most of the low-molecular-weight surfactants such as sodium lauryl sulfate (2.3 g/l) [16]. This suggests a strong tendency of the copolymers toward self-assembly of micelles. The low CMC also demonstrates the thermodynamic stability of the micelles and the prevention of premature drug release from micelles [17].

The copolymers would be hydrated below the LCST due to the production of hydrogen binding between the copolymers and water. The PNiPAAm moiety becomes lipophilic above the LCST because of the lipophilic interaction among the isopropyl groups. This leads to the fusion of the micelles. The size enlargement of the micelles as a function of temperature can confirm the production of aggregates. The hydrophilicity level of PNiPAAm can largely influence the transition temperature of the thermosensitive copolymers. Previous studies [9, 18] indicate that the LCST of PNiPAAm-*b*-PCL-*b*-PNiPAAm triblock copolymers increases with the increase in the chain length of the PNiPAAm block, which is due to the increased hydrophilicity of the copolymers. Our LCST results correlated with this tendency. PNiPAAm14-*b*-PCL59 showed an LCST value of 40°C. The increment of the PNiPAAm-based copolymer molecular weight and the degree of polymerization leads to the shift of the LCST to a higher temperature [15, 19]. The diameter of PNiPAAm8-*b*-PCL20 micelles increased to 567 nm at 40°C due to the aggregation. This size is still acceptable in the bloodstream without the risk of clogging. There would be finite aggregation of the micelles in a dilute system [20].

With a copolymer concentration prepared at 50-fold CMC, the copolymer with the largest PCL block (PNiPAAm14-*b*-PCL59) displayed the greatest micelle size (199 nm). The extension of the average diameter with increasing PCL length originates from the increment of lipophilic degree and the capacity in the micellar core [10, 21]. Nevertheless, this cannot explain the larger size of PNiPAAm9-*b*-PCL15 compared to PNiPAAm8-*b*-PCL20. This could be attributed to the longer length of the PNiPAAm block having the more-hydrated and bulkier shell [6, 16].

The hydrophilicity of the shell of PNiPAAm-based micelles has been found to be weakened once the temperature is increased above the LCST [17, 22]. This can result in the partial destruction of the micellar structure, leading to the facile drug release at body temperature. This was not the case in this study since PNiPAAm-*b*-PCL micelles exhibited a slower drug release upon heating to 37°C. The burst carboplatin release from the control solution was also restrained after incorporation into the micelles. Choi et al. [10] demonstrated that the phase transition causes a sol to gel transformation of PNiPAAm-*b*-PCL copolymers. An additional gel layer around the particulate surface is created above the LCST, contributing to the diffusion barrier for drug passage. It is postulated that the PNiPAAm block turns lipophilic above the LCST. This phase transition causes a thickening of the lipophilic segment in the micellar shell [23]. The drug-release process from the micelles includes the drug escape from the micellar core, and then the diffusion across the shell and the drug partitioning into the external phase occurs. The enlarged lipophilic shell prevents drug delivery into the aqueous phase and promotes drug entrapment in the micelles [20]. Almost no further carboplatin release was shown after an initial burst. Carboplatin inclusion in the micelles produced a prolonged and continuous release. This suggests a sustained delivery of carboplatin by micellar entrapment. This effect is especially important when administering the nanocarriers into circulation. The biodegradable copolymers always suffer from hydrolytic and enzymatic erosion in systemic circulation [24]. This may lead to a quick drug release before targeting the specific organs. PNiPAAm-*b*-PCL micelles may ameliorate this drawback.

Free DiR exhibited an extensive distribution in RES [25]. Our in vivo bioimaging profiles confirmed this trend. The RES uptake accelerated elimination in the body. DiR accommodated in micelles had avoided the recognition by RES, especially in the spleen. The nanoparticle size of <200 nm minimizes the degree of RES uptake and lessens renal excretion, thus increasing the possible targeting to specific organs or tumors [26]. The micelles developed in this study fulfilled this criterion. The thermosensitive micelles containing DiR still showed a significant accumulation in the liver. This was because of the critical role of the liver in PCL-based micelle clearance [27]. The micelles showed a negligible delivery to the brain, indicating a difficult passage across the blood–brain barrier (BBB). A significant accumulation of micelles was found in the lungs, demonstrating a passive targeting to this organ. It is proposed that the prolonged circulation of the micelles due to RES bypassing leads to an opportunity to target specific organs such as the lungs. The lungs serve in the circulation as the first filter for xenobiotics [28]. This effect is unlikely to be mediated by RES. Pulmonary vasculature contains extensive vascular networks with 25–30% of the total endothelial surface of the body [29]. The unique feature promotes the PNiPAAm-*b*-PCL micelle deposition in the lungs. The transition from the hydrophilic to the lipophilic corona of thermosensitive micelles at body temperature also accelerates micellar escape from the circulation to the peripheral tissues.

Since the DiR-loaded micelles largely accumulated in the lungs, the nanocarriers delivered a higher carboplatin amount to this organ as compared to the control. Micellar loading partly decreased the RES uptake of DiR. However, this effect was minor for carboplatin. It is interpreted that DiR and carboplatin are different molecules with different metabolism pathways. The tendency to escape RES by micelles is dependent on the loaded molecules. The thermoresponsive micelles enhanced carboplatin uptake by the lungs but decreased the distribution in the heart and kidneys. This approach ameliorated drug delivery to targeted tissues and reduced accumulation to nontargeted tissues, an improvement that would help to avoid side effects. A major problem of anticancer drug administration is the clearance of the renal system [30]. Free carboplatin is a small molecule with a molecular mass of 371 Da. Nanoparticles with a size of 10–200 nm can prevent the rapid renal excretion of some anticancer drugs [31]. The negative surface charge of micelles is beneficial in the blood for evading micellar aggregation caused by adsorption of plasma proteins [32]. The copolymer micelles with a very low CMC demonstrate an excellent stability in the systemic circulation owing to slower micelle dissociation than occurs with small molecular surfactant nanoparticles [11]. The physicochemical characteristics of PNiPAAm-*b*-PCL micelles suggest that this thermosensitive nanocarrier is stable in the circulation, making it conducive to slowly delivering the drug over a long period.

The experimental results suggested a passive targeting of PNiPAAm-*b*-PCL micelles to the lungs, which was preferred for pulmonary disease or lung cancer therapy. Active targeting such as antibody or peptide conjugation to nanoparticles is an efficient way to deliver the drug to targeted sites; nevertheless, the antibody-ligand nanocarriers are accompanied by a toxicity concern [33, 34]. For example, the exogenous antibodies can contribute to antibody-mediated acute lung injury [35]. The cost of the antibody is also high. Passive targeting by micelles is another option for efficient drug delivery to the lungs. The micelles exhibited no toxicity toward mammalian cells, including 293T and HaCaT. The results of the LDH assay demonstrated a favorable blood compatibility of micelles. The micelles developed in the present study can be regarded as safe. The next investigation for evaluating the applicability of the carboplatin-loaded micelles is the evaluation of tumor targeting and anticancer efficacy in the lungs.

## Conclusion

Diblock copolymer PNiPAAm-*b*-PCL was synthesized and characterized with the aim of combining thermosensitivity and biocompatibility. The grafted copolymers self-assembled into spherical micelles with an LCST of 33–40°C. The CMC of the copolymers was in the range of 1.8–3.5 mg/l, which decreased as the length of the PNiPAAm chains grew. The micelles formed a gel layer on the shell above the LCST, producing a delayed and sustained diffusion of carboplatin. We assessed neutrophil, 293T, and HaCaT cytotoxicity in response to the micelles, and we detected no toxicity to these cells. The improved lung delivery and decreased RES uptake of the micelles enabled passive targeting to the lungs. The avoidance of the undesirable organ accumulation and toxicity associated with the drug was also achieved. The amphiphilic copolymer micelles fabricated in this study appear promising as nanocarriers for carboplatin.

## Methods

### Materials

*N*-Isopropylacrylamide (NiPAAm), ε-caprolactone (CL), carboplatin, azobisisobutyronitrile (AIBN), and toluene were purchased from Sigma-Aldrich (St. Louis, MO, USA). 1,1′-Dioctadecyl-3,3,3′,3′-tetramethylindotricarbocyanine iodide (DiR) was supplied by ATT Bioquest (Sunnyvale, CA, USA). Benzoyl peroxide (BPO) came from Shimakyu (Osaka, Japan). 2-Mercaptoethanol was provided by Avantor (Center Valley, PA, USA). *N*,*N*-dimethylformamide (DMF) and tetrahydrofuran (THF) were purchased from Mallinckrodt (St. Louis, MO, USA). Tin(II) 2-ethylhexanoate (Sn(Oct)_2_) was from Strem Chemicals (Newburyport, MA, USA).

### Synthesis of PNiPAAm

A determined amount of NiPAAm (6 g for PNiPAAm8-*b*-PCL20, 4 g for PNiPAAm9-*b*-PCL15, and 2 g for PNiPAAm14-*b*-PCL59), 2-mercaptoethanol (0.2 g for PNiPAAm8-*b*-PCL20, 0.09 g for PNiPAAm9-*b*-PCL15, and 0.13 g for PNiPAAm14-*b*-PCL59), and 2% (w/v) BPO were added to a round-bottom flask under nitrogen atmosphere. THF (15 ml) was then added to the flask with stirring for 24 h. The crude product was purified by ether in the presence of THF to remove the unreacted monomer and initiators. The product was purified twice to obtain the white powder of PNiPAAm. Figure 1 shows the scheme of PNiPAAm synthesis.

### Synthesis of PNiPAAm-b-PCL

The diblock copolymer of PNiPAAm-*b*-PCL was prepared through a ring-opening polymerization of CL with PNiPAAm in toluene by utilizing Sn(Oct)_2_ as the catalyst. PNiPAAm (5.92 g for PNiPAAm8-*b*-PCL20, 5.1 g for PNiPAAm9-*b*-PCL15, and 5.0 g for PNiPAAm14-*b*-PCL59), CL (10.3 g for PNiPAAm8-*b*-PCL20, 9.1 g for PNiPAAm9-*b*-PCL15, and 20.1 g for PNiPAAm14-*b*-PCL59), and 1.5% (w/v) Sn(Oct)_2_ were refluxed in toluene (30 ml) under a nitrogen stream for 24 h. After this reaction, the mixture was concentrated by rotary evaporator. The product was dissolved in chloroform and precipitated into ether/*n*-hexane (1:5). This procedure was repeated twice. The purified copolymer was dried under vacuum conditions. Figure 1 demonstrates the process of the copolymer synthesis.

### ^1^H nuclear magnetic resonance (^1^H NMR)

^1^H nuclear magnetic resonance spectra were detected by a Bruker Avance 400 MHz spectrometer (Billerica, MA, USA) with CDCl_3_ as a solvent.

### Fourier transform infrared (FTIR) spectroscopy

Fourier transform infrared spectra of the polymers and copolymers were recorded using a Bruker Tensor 27 FTIR spectrophotometer. The samples were pressed into KBr pellets for analysis.

### Molecular weight

The molecular weights of the copolymers were measured by gel permeation chromatography (GPC), which was performed on a Jasco high-performance liquid chromatography (HPLC) system (Tokyo, Japan) equipped with a refractive-index detector. Jordi Gel polydivinyl benzene columns with pore sizes of 100, 500, and 1,000 Å were employed for determination. The eluent used in this experiment was chloroform at a flow rate of 0.5 ml/min. Polyethylene glycol was used as the standard for establishing a calibration curve.

### Preparation of micelles

The copolymer micelles of PNiPAAm-*b*-PCL were prepared using the dialysis method. A solution of diblock copolymer (30 mg) was dissolved in DMF (5 ml) and was then placed in a dialysis bag with a molecular weight cutoff of 3,500 Da. Carboplatin (5 mg) was added in DMF to prepare the drug-containing nanocarriers if necessary. The DMF solution was dialyzed against double-distilled water (ddH_2_O) at room temperature for 24 h. The water was replaced every 30 min during the first 3 h.

### Critical micelle concentration (CMC)

The CMC of the copolymers was measured by fluorescence spectroscopy (Hitachi F-7000, Tokyo, Japan) using pyrene as the probe. The pyrene solution (100 μl) in acetone at a concentration 6.1 × 10^−5^ M was added to a vial. After evaporation, the measured quantities of micellar solutions with various concentrations of PNiPAAm-*b*-PCL ranging from 1.83 × 10^−5^ to 0.3 g/l were incorporated into the vial. The concentration of pyrene in the micellar solution was 6.1 × 10^−7^ M. After equilibration at room temperature overnight, the fluorescence excitation spectra of the final solution were determined at an emission wavelength of 390 nm. The excitation fluorescence values *I*_334_ and *I*_331_, respectively, at 334 and 331 nm, were detected for the subsequent measurement of the CMC.

### Lower critical solution temperature (LCST)

The LCST of the diblock copolymers was measured by detecting the optical transmittance. A UV spectrometer (Jasco V-550, Tokyo, Japan) was used by monitoring the transmittance of a 500-nm light beam through the copolymer solution at the concentration of a 50-fold CMC. The samples were heated at a rate of 0.15°C/min from 25 to 50°C. The LCST was determined at the temperature at which the optical transmittance was 50%.

### Average diameter and zeta potential

The mean diameter (*z*-average) and zeta potential of the prepared micelles were detected using a laser-scattering method (Nano ZS90, Malvern, Worcestershire, UK). The determination was performed at the concentration of a 50-fold CMC.

### Transmission electron microscopy (TEM)

The morphology of PNiPAAm-*b*-PCL micelles was monitored by Jeol JEM-1400 TEM (Tokyo, Japan). A 10-μl of micelle dispersion was pipetted onto a carbon-film-coated copper grid to form a thin-film specimen and stained with 1% phosphotungstic acid. The prepared samples were photographed by TEM.

### Carboplatin release from micelles

This experiment was conducted with Franz diffusion cells. A cellulose membrane with a molecular weight cutoff of 6,000–8,000 Da (Cellu-Sep^®^ T2, Membrane Filtration Products, Seguin, TX, USA) was mounted between the receptor and the donor. The receptor contained 30% ethanol in pH 7.4 buffer with a volume of 5.5 ml. The donor (0.5 ml) was loaded with carboplatin-containing micelle dispersion or carboplatin in control solution (4% DMSO in double-distilled water). Carboplatin concentration in the dispersions was 0.1 mg/ml. The available diffusion area for carboplatin was 0.785 cm^2^. The temperature was set at 25 or 37°C. The 300 μl aliquots of the receptor medium were withdrawn at determined intervals. The receptor compartment was immediately added to an equal volume of fresh medium. The samples taken from the receptor were analyzed by atomic absorption spectroscopy (Hitachi Z-5000, Tokyo, Japan) to quantify the amount of carboplatin.

### Cytotoxicity determined by lactate dehydrogenase (LDH)

The neutrophils from healthy volunteers (20–30 years old) were purified using a protocol approved by the Institutional Review Board at Chang Gung Memorial Hospital. All subjects were required to provide written informed consent. Neutrophils were isolated with sedimentation prior to centrifugation in a Ficoll Hypaque gradient and hypotonic lysis of erythrocytes. LDH leakage from the neutrophils after micelle treatment was detected by a commercially available method (CytoTox 96^®^, Promega, Madison, WI, USA). Neutrophils (6 × 10^5^ cells/ml) were equilibrated at 37°C for 2 min and subsequently treated with copolymer dispersion at different concentrations (1.5–48 μg/ml) for 15 min. The cytotoxicity was displayed by LDH release in cell-free medium as a percentage of the total LDH released. The total LDH release was measured by treating 0.1% Triton X-100.

### Cell viability

Human embryonic kidney cells (293T) and human keratinocytes (HaCaT) were seeded in 96-well plates by properly-selected culture medium for 24 h. Different volumes of copolymer dispersion were added to the wells to make final copolymer concentrations of 1.5, 3, 6, 12, and 48 μg/ml. The incubation time of the cells exposed to the micelles was 24 h. Tetrazolium salt in isopropanol was pipetted into the wells after 24 h. The optical density of the dissolved material was determined using a spectrophotometer to calculate the viability of the 293T and HaCaT cells.

### Animals

Male Sprague–Dawley rats (~400 g) were used for the in vivo experiments. The experimental protocol was received and approved by the Institutional Animal Care and Use Committee of Chang Gung University. All animals were housed and handled according to the institutional guidelines.

### Organ bioimaging

The rats were anesthetized using Zoletil^®^ 50 (60 mg/kg). The body temperature of the rats was kept at 37°C with a heating pad. The control solution or micelle dispersion containing DiR (0.05%, w/v) as a fluorescence dye was injected into the femoral vein at a volume of 0.25 ml/kg. Isoflurane/oxygen was used to maintain the anesthetized status. The rats were sacrificed 2 h postinjection. The organs were removed from the body and washed with saline. The DiR fluorescence in the organs was monitored using the Pearl^®^ Impulse imaging system (Li-Cor, Lincoln, NE, USA) at near IR wavelength.

### Biodistribution of carboplatin

Twelve rats were randomly divided into two groups. The rats in each group were injected with the control solution (4% DMSO in ddH_2_O) or micelles at a carboplatin dose of 0.25 mg/kg. The rats were sacrificed 2 h after dosing. The brain, lungs, heart, liver, spleen, and kidneys were harvested from the body. The organs were weighed and suspended in methanol (1 ml) by MagNA Lyser (Roche, Indianapolis, IN, USA). The homogenates were centrifuged at 10,000×*g* for 10 min. The supernatant (0.2 ml) was taken and then mixed with acetonitrile (0.6 ml). Following a centrifugation at 10,000×*g* for 10 min, the supernatants were assessed by atomic absorption spectroscopy.

### Statistical analysis

Statistical calculation was performed using an unpaired *t*-test. The post hoc Newman–Keuls test was utilized to check the individual differences between the groups. A probability of <0.05 was considered significant.

## Additional file

**Additional file 1.** Physicochemical characterization of PNiPAAm-*b*-PCL micelles and the carboplatin accumulation (μg/mg) in organs after intravenous administration of solution vehicle and PNiPAAm8-*b*-PCL20 micelles.
