# Supplementary material for: Passive targeting of thermosensitive diblock copolymer micelles to the lungs: synthesis and characterization of poly(N-isopropylacrylamide)-block-poly(ε-caprolactone)
Source: J Nanobiotechnology. 2015 Jun 18;13:42. doi: 10.1186/s12951-015-0103-7 (PMC4472254; doi:10.1186/s12951-015-0103-7)
Supplement: Supplementary file 1 — Additional file 1. Physicochemical characterization of PNiPAAm-b-PCL micelles and the carboplatin accumulation (μg/mg) in organs after intravenous administration of solution vehicle and PNiPAAm8-b-PCL20 micelles. [file 12951_2015_103_MOESM1_ESM.docx]

Table 1. Physicochemical characterization of PNiPAAm-*b*-PCL micelles

| Copolymer | MW_GPC_ (Da) | CMC (mg/l) | Size (nm) | Zeta potential (mV) | LCST (°C) |
| --- | --- | --- | --- | --- | --- |
| PNiPAAm8-*b*-PCL20 | 5578 | 3.46 | 145.8±18.3 | -17.6±11.6 | 33 |
| PNiPAAm9-*b*-PCL15 | 3792 | 2.14 | 166.5±8.2 | -29.8±0.1 | 35 |
| PNiPAAm14-*b*-PCL59 | 6879 | 1.75 | 198.6±14.7 | -26.8±0.5 | 40 |

MW_GPC_, the molecular weight measured by gel permeation chromatography.

CMC, critical micelle concentration.

LCST, lower critical solution temperature.

Table 2. The size, polydispersity, and zeta potential of PNiPAAm8-*b*-PCL20 at different temperatures

| Temperature (°C) | Size (nm) | Polydispersity | Zeta potential (mV) |
| --- | --- | --- | --- |
| 25 | 145.8±18.3 | 0.15±0.14 | -17.6±11.6 |
| 36 | 369.6±22.9 | 0.10±0.03 | -14.8±3.6 |
| 40 | 566.8±30.3 | 1.00±0.00 | -15.9±3.9 |

Each value represents the mean and standard deviation (*n*=3).

Table 3. Carboplatin accumulation (μg/mg) in organs after intravenous administration of solution vehicle and PNiPAAm8-*b*-PCL20 micelles

| Organ | Solution | Micelles |
| --- | --- | --- |
| Brain | 0.81±0.27 | 0.81±0.25 |
| Lung | 2.50±0.74 | 3.35±0.68* |
| Heart | 1.83±0.66 | 1.04±0.18* |
| Liver | 0.44±0.11 | 0.51±0.14 |
| Spleen | 2.10±0.27 | 2.20±0.38 |
| Kidney | 1.45±0.53 | 0.74±0.12* |

*, *p* < 0.05 as compared to the data of control solution.

Each value represents the mean and standard deviation (*n*=6).
